# Influences of Enzyme Blend Supplementation on Growth Performance, Nutrient Digestibility, Fecal Microbiota and Meat-Quality in Grower-Finisher Pigs

**DOI:** 10.3390/ani10030386

**Published:** 2020-02-27

**Authors:** Balamuralikrishnan Balasubramanian, Jae Hong Park, Sureshkumar Shanmugam, In Ho Kim

**Affiliations:** 1 Department of Animal Resources and Science, Dankook University, Cheonan 31116, Korea; geneticsmurali@gmail.com (B.B.); parkjh@dankook.ac.kr (J.H.P.); sureshbiogenetic@gmail.com (S.S.); 2 Department of Food Science and Biotechnology, College of Life Science, Sejong University, Seoul 05006, Korea

**Keywords:** enzyme blend, meat quality, nutrient digestibility, growth performance, fecal microbiota

## Abstract

**Simple Summary:**

In livestock nutrition, wide use of antibiotics leads to antibiotic resistance that can have an adverse impact on animal health. For this reason, various feed additives have been used as alternatives to growth promotors to improve animal performance. This study evaluates the effects of enzyme blend supplementation on the performance of pigs. The results demonstrated that dietary inclusion of an enzyme blend improved the growth performance, digestibility, meat quality and microbial populations in pigs. These findings are useful to the development of new feed additives in the livestock industry.

**Abstract:**

The study was aimed to evaluate the effects of dietary inclusion of an enzyme blend on growth performance, apparent total track digestibility (ATTD) of dry matter (DM), nitrogen (N), gross energy (GE), fecal microbial population, noxious gas emissions and meat quality of pigs fed corn–soybean meal-based diets for a 16-week feeding trial. A total of 180 growing pigs (body weight of 23.3 ± 2.51 kg) were used and randomly allotted to one of three dietary treatments (positive control (PC, basal diet); negative control (NC, −150 kcal/kg of PC); A1 (NC + 1% enzyme blend)). Overall, dietary inclusion of the enzyme blend increased (*p* < 0.05) body weight, average daily gain and gain:feed ratio without effecting average daily feed intake. An increase was observed in ATTD of DM (*p* = 0.027) and GE (*p* = 0.026) at week 16 and 6, respectively. Dietary inclusion of the enzyme blend increased the beneficial effects on fecal microbiota counts such as *Lactobacillus* with a reduced presence of *E. coli* during the entire experiment (*p* < 0.05). Further, positive effects (*p* < 0.05) were observed on back-fat thickness and carcass weight of pigs, along with the results of reduced levels of NH_3_ emissions (*p* = 0.032) at week 16. Thus, the study suggested that the dietary enzyme blend supplement had improving effects on growth performance, ATTD of nutrients, fecal microbial counts and meat quality in pigs.

## 1. Introduction

Total production costs in the swine-based industry have largely corresponded to the feed costs, making it lose out on nearly 70% of profits [1]. The energy content of the basal diet is a major determinant of pig performance and is the most expensive part of the diet’s cost. Corn–soybean meal (SBM)-based diets are both common energy and protein sources for swine diets in South Korea. The non-starch polysaccharides (NSP) in corn–SBM-based diets can negatively affect the performance, which in turn can have serious consequences for the profitability of the pork industry [2,3]. Corn contains 0.9% soluble and 6% insoluble NSP, while soybean contains 6% soluble and 18% to 21% insoluble NSP [4,5]. Therefore, an increasing consideration is paid on enzyme utilization in livestock nutrition. Exogenous enzyme supplementation is used to target NSP and protein, consequently improving digestion, weight gain in monogastric animals fed corn–SBM diets [6,7] and absorption of nutrients such as energy and protein, while reducing feed costs [8]. Increasing dietary energy from added fat has been consistently shown to be able to improve growth performance and feed efficiency from the middle to late nursery period. However, with increased cost of added fat, alternatives are being sought to increase energy density at lower cost. Cost-cutting alternatives such as inclusion of natural by-products in the animal diets have also become a reality. According to the studies of Whitney et al. [9] and Ying et al. [10,11] enzyme-based liquid supplementation can improve the growth performance of pigs. Limited research studies have determined the effects of enzyme-based liquid energy in diets of growing–finishing pigs. Therefore, the present study was conducted to examine the effects of dietary inclusion of an enzyme blend on growth performance, fecal microbiota, apparent total tract digestibility (ATTD), excreta gas emissions and meat quality of grower–finisher pigs.

## 2. Materials and Methods

### 2.1. Source of Feed Additive and Animal Ethics

In this study, a commercial product (Alcopro^®^, Simco Nutrition Group^™^, Irvine, California, CA, USA) containing about 10,000 kcal/kg metabolizable energy (ME), high energy source ingredients (corn distillers condensed soluble and ethyl alcohol) and a natural digestive enzyme blend (glucoamylase from *Aspergillus niger*, alpha-amylase from *Bacillus stearothermophilos*, lipase, maltase, cellulose, protease) was used. The level of energy supplementation was based on the recommendations of the manufacturer. The product was not oxidized or rancid and was a stable liquid in storage. The experimental protocol (DK-634) used in the present study was approved by the Animal Care and Use Committee of Dankook University, Cheonan, South Korea.

### 2.2. Experimental Design, Animals, Housing and Diets

One hundred and eighty ((Landrace × Yorkshire) × Duroc) pigs with an initial body weight (BW) of 23.3 ± 1.40 kg was used for a 16-week feeding trial. Pigs were allocated to one of three dietary treatments: positive control (PC, basal diet); negative control (NC, −150 kcal/kg of PC); A1 (NC + 1% enzyme blend). Each treatment consisted of twelve replications with five pigs (3 gilts and 2 castrated barrows) per pen in a randomly complete block design based on gender and BW. Diet in mash form was formulated to meet or exceed the nutritional requirements of pigs, according to National Research Council [12] recommendations for nutrient requirements of swine (Table 1). These dietary treatments were given during grower (0–6 weeks) and finisher (7–16 weeks) phases. Pigs were housed in an environmentally controlled system, and each pen was equipped to allow ad libitum access to feed and water throughout the experimental period.

### 2.3. Sampling and Measurements

Pigs were weighed at the start and at week 2, 6, 8, 12 and 16 of the experimental periods, and feed consumption was recorded throughout the experiment to calculate average daily gain (ADG), average daily feed intake (ADFI) and gain:feed ratio (G:F). Chromic oxide (2 g kg^−1^) was added to the diet as an indigestible marker to allow ATTD determinations of dry mater (DM), as previously described [13,14]. Nitrogen (N) was determined by a Kjectec 2300 nitrogen analyzer (Foss Tecator AB, Hoeganaes, Sweden), and crude protein (CP) was calculated as nitrogen × 6.25. Gross energy (GE) was determined by using a Parr 6100 oxygen bomb calorimeter (Parr Instrument Co., Moline, Illinois, USA). Dietary DM (method 930.15), crude protein (method 968.06), crude fat (991.36), crude fiber (992.16), crude ash (942.05), calcium (method 984.01) and phosphorus (method 965.17) were analyzed according to the procedures described by AOAC [13].

Fresh fecal samples were directly collected via rectal massage of two pigs in each pen at 6 and 16 weeks of the experiment to determine the fecal microbial counts. One gram of composite fecal sample from each pen was diluted with 9 mL of 1% peptone broth (Becton, Dickinson and Co., Franklin Lakes, NJ) and homogenized. Viable counts of bacteria in fecal samples were determined by plating serial 10-fold dilutions (in 1% peptone solution) onto MacConkey agar plates (Difco Laboratories, Detroit, MI) and *Lactobacilli* medium III agar plates (Medium 638, DSMZ, Braunschweig, Germany) to isolate *Escherichia coli* and *Lactobacillus*, respectively. *Lactobacilli* medium III agar plates were incubated at 39 °C for 48 h under anaerobic conditions. MacConkey agar plates were incubated at 37 °C for 24 h. The numbers of *E. coli* or *Lactobacillus* colonies were counted immediately after plates were removed from the incubator [15]. The microbial populations were log transformed before statistical analysis.

The NH_3_ concentration was then determined using the method described by Chaney and Marbach [16]. To determine the fecal H_2_S and total mercaptans (R.SH) concentration, 300 g of fresh fecal samples were transferred to a sealed box and fermented in an incubator for 30 h (35 °C). The fermented samples were then analyzed with a gas search probe (Gastec Model GV-100, detector tube No. 4LL, 4LK for H2S; No.70 and 70 L for R.SH, Gastec Corp., Kanagawa, Japan) [17].

At the end of the experiment, pigs were slaughtered at a local commercial slaughterhouse when they reached an average BW of 110 kg. Carcasses were chilled at 2 °C for 24 h. A sample of the right loin was obtained between the 10^th^ and 11^th^ ribs. Meat samples were thawed at 26 °C before evaluation. Sensory evaluation (color, marbling and firmness scores) was conducted on the 10^th^-rib chop according to NPPC [18] standards at 26 °C. Color, marbling and firmness were scored by a sensory panel using a five-point scale (1 = pale, devoid of marbling, very soft; 5 = dark, moderately abundant marbling or greater, very firm). The sensory panel was comprised of 10 panelists, all of whom were trained to evaluate the sensory attributes of color, marbling and firmness [18]. Immediately after collection of chops, values for L (lightness = 89.2), a (redness = 0.921) and b (yellowness = 0.783) were obtained from three orientations on the 10^th^-rib chop using a Model CR-410 chromameter (Konica Minolta Sensing Inc., Osaka, Japan) of CIE (Commission Internationale de L’Eclairage) and Hunter. The color was measured on each loin meat sample in duplicate with one reading in the anterior and one reading in the posterior portion of the meat. All color readings were taken on the skin side surface in an area free of obvious color defects (over scald, bruises and blood accumulation). At the same time, duplicate pH values of each sample were directly measured using a pH meter (Istek, Model77p). Longissimus muscle area (LMA) and back-fat thickness (BFT) were measured by tracing the LM surface at the 10th rib using the aforementioned digitizing area–line sensor. The water-holding capacity (WHC) was measured using the method of Kauffman et al. [19]. The carcass back-fat thickness (BFT) was adjusted to a live weight of 110 kg; drip loss of approximately 3 g of meat sample was measured using the plastic bag method, and cook loss was determined as described by Honikel [20].

### 2.4. Statistical Analysis

All data were statistically analyzed by analysis of variance, using the general linear model procedure of SAS/STAT^®^ 9.2 (SAS Inst. Inc., Cary, NC) with a complete randomized block design; each pen served as the experimental unit. Variability in data was expressed as pooled standard error of means. Differences among treatment means were determined using Turkey’s range test. Differences were deemed significant when *p* ≤ 0.05, and trends were noted when 0.05 < *p* < 0.10.

## 3. Results and Discussion

The hypothesis of the study was that the potential to increase the dietary supplement in corn-based soy bean meal may contribute to improvement of growth performance, meat quality and carcass grades in grower–finisher pigs. The present study revealed that dietary inclusion of an enzyme blend resulted in a tendency of increased BW at week 6 (*p* = 0.080) and a significant increase at week 16 (*p* = 0.038). Dietary enzyme blend supplementation had a significant difference on ADFI and G:F ratio (*p* = 0.025, 0.011, respectively) and tendentially increased ADG (*p* = 0.071) during the grower phase (Table 2). Our findings are line with Ying et al. [10] who reported that diets of nursery pigs supplemented with a liquid feed additive with choice white grease could significantly improve the ADG and ADFI G:F ratio. Likewise, improvement of the digestion and ADG of monogastric animals fed corn–SBM-based diets have been reported by the use of enzymes such as xylanase [7,21,22], amylase and protease [23]. Similarly, Whitney et al. [9] discussed improved growth performance among grower–finisher pigs fed with diets supplemented with corn distillers dried grain with solubles sourced from an ethanol plant, in accordance with our reported results.

On the contrary, a previous study reported that the diets supplemented with a liquid feed additive containing enzymes did not have significant effects on the growth performance of nursery pigs [11]. Enzyme-based applications in corn and SBM-based diets have yielded beneficial effects on piglets [24]. These inconsistent responses to alcohol-based liquid feed supplementation in pig diets may be due to the fact that experimental animals used in these studies varied in age, health status, breed and supplementation content. Observed results showed that the dietary enzyme blend inclusion led to a higher ADG (*p* = 0.002; 0.033) and G:F ratio (*p* = 0.002; 0.004), respectively, at week 16, and overall, without effects on ADFI compared with other treatments. Such a conclusion can be attributed to the low energy composition of the diet, which is in agreement with an earlier report, which concluded that diets having a difference in energy content of less than 124 kcal can be considered uninfluential parameters for feed intake [25]. Xylanases have been a preferred choice for enhancing nutrient digestibility due to their advantages such as enabling access to trapped nutrients to digestive enzymes and their action of cell wall degradation [26]. The objective of this study includes the assessment of the potential effects of an enzyme blend to improve nutrient digestibility of corn–SBM-based diets in pigs. In our study, dietary enzyme blend supplementation indicated a higher ATTD of GE (*p* = 0.026) and DM (*p* = 0.027) at week 6 and 16, respectively, which is in line with Li et al. [19]. However, there were no significant effects on ATTD of N in the entire experiment (Table 3). Our results showed an increase in DM digestibility on inclusion of dietary enzyme blend inclusion in accordance with the previous studies [7,23]. These studies showed supplementation of an enzyme blend to a corn–SBM-based diet increased apparent digestibility and growth performance in pigs.

The present study indicates supplementation of diet with enzyme blend has beneficial effects on fecal microbiota in grower–finisher pigs. The effects of fecal microbial counts were reflected by increased fecal *Lactobacillus* (*p* = 0.048, 0.012) and reduced *E. coli* counts (*p* = 0.043, 0.063) relative to other diets at week 6 and 16 (Table 4). An upsurge in introducing more microbiota in order to enhance digestibility and health conditions of the gut has been discussed previously [11]. A relation was also observed between the digestibility and gut health with fecal noxious gas content [7,26,27,28] because increased digestibility may allow less substrate for the microbial fermentation in the large intestine, which consequently decreases the fecal noxious gas content. Similarly, inclusion of dietary enzyme blend led to lower fecal NH_3_ (*p* = 0.033) content when compared to control diet at end of the experiment without differences on H_2_S and total mercaptans (Table 4). Pigs fed diets supplemented with an enzyme blend showed higher carcass weight (*p* = 0.005), reduced BFT (*p* = 0.009) and tendential effects on cooking loss (*p* = 0.061) and color of lightness (*p* = 0.094), as seen in Table 5. Furthermore, the study showed increased sensory evaluation of color and marbling, although the differences were not statistically significant. Statistically insignificant differences were observed in drip loss, pH, LMA and WHC in the current study (Table 5). Based on previous studies [17,21,28,29] different meat quality parameters such as pH value and meat color that corelate with our results may help us infer or conclude our observations to a greater extent.

## 4. Conclusions

Although enzyme blend supplementation had positive effects on the growth performance of grower–finisher pigs, the absence of positive effects by the supplemented enzyme blend on meat quality traits indicated that nutrient utilization was not significantly improved. Further research is needed to determine if an enzyme blend supplementation of energy can be found in grower–finisher pigs; the research for an effective enzyme blend supplement most likely will continue in future.

## Figures and Tables

**Table 1:** Ingredients and composition of basal diets for grower–finisher pigs (g/kg, as-fed basis).

Item	Positive Control		Negative Control (ME −150 kcal)	
	Grower	Finisher	Grower	Finisher
Ingredient				
Corn	585.8	703.4	541.6	656.2
Oat	50.0	50.0	10.0	10.0
Molasses	31.00	10.0	30.0	10.0
Soybean meal (CP, 48%)	261.8	180.00	244.9	170.0
Rapeseed meal	16.0	-	15.0	-
Lysine (78%)	0.50	1.8	0.30	1.6
Tallow(liquid)	30.1	30.0	27.4	27.4
Limestone	7.9	7.9	7.9	7.9
Dicalcium phosphate	11.8	11.8	11.8	11.8
Salt	2.00	2.00	2.00	2.00
Vit. premix ^A^	2.00	2.00	2.00	2.00
Mineral premix ^B^	1.00	1.00	1.00	1.00
Choline	0.10	0.1	0.10	0.1
Calculated composition				
ME, kcal/kg	3336	3349	3186	3199
Analyzed composition				
Crude protein	165.3	143.6	157.5	136.5
Crude fat	5.88	6.15	5.95	6.23
Crude fiber	3.27	2.89	3.30	2.92
Crude ash	4.88	4.27	4.87	4.28
Calcium	6.8	6.3	6.8	6.4
Total phosphorous	5.5	5.2	5.4	5.2
Available lysine	7.8	6.9	7.5	6.7
Available methionine	2.1	1.8	2.0	1.7

^A^ Provided per kilogram of diet: vitamin A—4.5 mg, vitamin D_3_—0.0935 mg, vitamin E—37.5 mg, vitamin K_3_—2.55 mg, vitamin B_1_—3 mg, vitamin B_2_—7.5 mg, vitamin B_6_—4.5 mg, vitamin B_12_—0.024 mg, vitamin B_3_—51 mg, vitamin B_9_—1.5 mg, vitamin B_7_—126 mg, vitamin B_5_—13.5 mg. ^B^ Provided per kilogram of diet: Zn (ZnSO_4_)—37.5 mg, Mn (MnO_2_)—137.5 mg, Fe (FeSO_4_∙7H_2_O)—37.5 mg, I (KI)—0.83 mg, Se (Na_2_SeO_3_∙5H_2_O)—0.23 mg.

**Table 2:** Effects of dietary supplementation of enzyme blends on growth performance traits in grower–finisher pigs.

Traits	PC	NC	A1	SEM	*p*-Value
Body weight, kg—Grower phase					
Initial	23.1	23.05	22.99	0.07	0.532
Week 2	32.83	32.56	32.65	0.14	0.415
Week 6	49.12 ^b^	50.5 ^a^	50.32 ^ab^	0.40	0.081
Body weight, kg—Finisher phase					
Week 8	61.89	61.39	62.32	1.14	0.846
Week 12	91.19	89.87	91.76	1.14	0.519
Week 16	112.20 ^ab^	106.9 4 ^b^	114.08 ^a^	1.65	0.038
Week 6—Grower Phase					
ADG (g)	620 ^b^	654 ^a^	651 ^ab^	10	0.072
ADFI (g)	1726 ^a^	1638 ^ab^	1526 ^b^	41	0.025
G:F	0.361 ^b^	0.399 ^a^	0.427 ^a^	0.011	0.011
Week 16—Finisher Phase					
ADG (g)	898 ^a^	813 ^b^	922 ^a^	15	0.002
ADFI (g)	2703	2774	2794	58	0.535
G:F	0.333 ^a^	0.294 ^b^	0.330 ^a^	0.006	0.002
Overall					
ADG (g)	796 ^ab^	749 ^b^	813 ^a^	14	0.034
ADFI (g)	2738	3028	2809	92	0.129
G:F	0.292 ^a^	0.248 ^b^	0.290 ^a^	0.007	0.004

PC: positive control; NC: negative control (−150 kcal/kg of PC); A1: NC + 1% enzyme blend; SEM: standard error of means; ADG: average daily gain; ADFI: average daily feed intake; G:F: gain:feed. ^a,b^ Means in the same row with different superscripts differ (*p* < 0.05).

**Table 3:** Effects of dietary supplementation of enzyme blend on apparent total tract digestibility of grower–finisher pigs.

Traits (%)	PC	NC	A1	SEM	*p*-Value
Dry matter					
Week 6	75.15	74.33	74.48	0.93	0.811
Week 16	71.71 ^b^	69.69 ^ab^	73.05 ^a^	0.69	0.027
Energy					
Week 6	73.95 ^b^	72.81 ^ab^	75.56 ^a^	0.57	0.026
Week 16	70	70.3	71.75	0.73	0.266
Nitrogen					
Week 6	74.37	72.09	73.65	1.10	0.217
Week 16	69.94	69.47	71.90	1.47	0.494

PC: positive control; NC: negative control (−150 kcal/kg of PC); A1: NC + 1% enzyme blend; SEM: standard error of means. ^a,b^ Means in the same row with different superscripts differ (*p* < 0.05).

**Table 4:** Effects of dietary supplementation of enzyme blend on fecal microflora and excreta–noxious gas emissions in grower–finisher pigs.

Items	PC	NC	A1	SEM	*p*-Value
Fecal microbial (log_10_ cfu/g)					
Week 6					
*Lactobacillus*	7.16 ^b^	7.07 ^b^	7.36 ^a^	0.05	0.048
*E. coli*	6.39 ^a^	6.35 ^ab^	6.26 ^b^	0.03	0.043
Week 16					
*Lactobacillus*	7.32 ^b^	7.30 ^b^	7.43 ^a^	0.03	0.012
*E. coli*	6.42	6.36	6.24	0.04	0.064
Excreta noxious gas emission (mg/kg)					
Week 6					
NH_3_	3.91	3.58	3.27	0.42	0.383
H_2_S	3.12	3.26	2.38	0.33	0.251
Total mercaptans	5.16	5.08	4.72	0.29	0.493
Week 16					
NH_3_	13.51 ^a^	11.83 ^ab^	10.74 ^b^	0.39	0.032
H_2_S	22.84	22.71	21.61	0.25	0.806
Total mercaptans	17.45	16.85	16.80	0.32	0.368

PC: positive control; NC: negative control (–150 kcal/kg of PC); A1: NC + 1% enzyme blend; SEM: standard error of means. ^a,b^ Means in the same row with different superscripts differ (*p* < 0.05).

**Table 5:** Effects of dietary supplementation of enzyme blend on meat quality of grower–finisher pigs.

Traits	PC	NC	A1	SEM	*p*-Value
Color					
L—Lightness	56.03	55.40	56.26	0.25	0.094
a—Redness	18.22	17.90	18.54	0.44	0.612
b—Yellowness	7.74	7.81	7.37	0.38	0.691
Sensory evaluation					
Color	2.48	2.56	2.64	0.08	0.426
Firmness	2.88	3.18	3.27	0.11	0.409
Marbling	1.3	1.2	1.4	0.16	0.698
Cooking loss (%)	27.60	27.81	26.15	0.98	0.061
Drip loss (%)					
Day 1	4.65	4.29	4.49	0.47	0.867
Day 3	9.03	9.36	9.11	0.65	0.930
Day 5	14.44	13.84	13.46	0.82	0.709
Day 7	20.2	19.47	20.38	0.81	0.710
pH	5.26	5.23	5.24	0.02	0.640
Loin muscle area (cm^2^)	47.01	48.27	45.35	1.06	0.191
Water holding capacity (%)	60.34	60.9	58.92	0.96	0.355
Carcass weight (kg)	88.48 ^ab^	87.18 ^b^	90.29 ^a^	0.41	0.005
Back-fat thickness (mm)	17.44 ^a^	18.1 ^a^	16.26 ^b^	0.21	0.009

PC: positive control; NC: negative control (−150 kcal/kg of PC); A1: NC + 1% enzyme blend; SEM: standard error of means. ^a,b^ Means in the same row with different superscripts differ (*p* < 0.05).
